# P23 Antibody negative Systemic Sclerosis

**DOI:** 10.1093/rap/rkac067.023

**Published:** 2022-09-28

**Authors:** Daniel Mills, Sarah R Moore

**Affiliations:** North Manchester General Hospital, Manchester, United Kingdom; North Manchester General Hospital, Manchester, United Kingdom

## Abstract

**Introduction/Background:**

This case explores some classic rheumatology topics - systemic sclerosis and antibodies. It is now well recognised that there are a magnitude of different antibodies, beside the typical anti-Scl-70, anticentromere or anti-RNA polymerase III, that can be associated with systemic sclerosis however in 5-10% of cases all antibody tests are negative, yet the patient still displays characteristic clinical features of the disease. We need to ensure such patients are correctly diagnosed, followed up and managed.

**Description/Method:**

This case looks at the uncommon presentation of systemic sclerosis without any positive antibody results. I consulted with a 53-year-old male with a past medical history of hypertension, type 2 diabetes, GORD, hypothyroidism, depression and hyperlipidaemia. He had previously been seen in the rheumatology clinic with costochondritis and Raynaud’s phenomenon. At this time, he had antibody tests which were all negative (ANA and ENA). He had nailfold capillaroscopy which showed some concerns with widening of capillaries in areas and some linear haemorrhages. This was felt to not be diagnostic of Systemic sclerosis but advised continued follow up and repeat tests. Unfortunately, he failed to attend a subsequent clinic appointment and was lost to follow up. He was referred back to the Rheumatology department in 2021 with worsening symptoms of Raynaud’s. At this time he also described symptoms of reflux and dysphagia. He was suffering from generalised fatigue and pains. On examination he had tightening of the skin on his hands and was prayer sign positive. His antibody tests were repeated, again all coming back negative. We then arranged for repeat nailfold capillaroscopy and thermographic testing. This showed very little rewarming after cold challenge with delay even at 30 degrees. The nailfold capillaroscopy showed generalised widening of the capillaries with occasional distortion and tortuosity. There were multiple linear haemorrhages. This study was reported showing definite abnormality in keeping with a diagnosis of systemic sclerosis.

**Discussion/Results:**

This highlights the rare but now well recognised phenomenon of antibody negative systemic sclerosis which makes up around 5-10% of cases. It also shows how nailfold capillaroscopy can be a key tool in diagnosing this, and the features to look out for in order to do so - capillary widening, haemorrhage and loss of architecture. The case demonstrates some of the more common features of antibody negative systemic sclerosis including a higher proportion of male patients and prominent GI features. There are generally less severe vascular features including pulmonary hypertension and telangiectasia, with Raynaud’s less often leading to digital ulcers and pitting. In this case we suggested uptitration of his nifedipine to try and help control his Raynaud’s symptoms. Given the significant GI features this patient was experiencing we referred him on for further evaluation by the gastroenterology team. I think some aspects of this case were blurred by his comorbidities, especially diabetes, and some of his skin disease and pains were attributed to this. However, with hindsight and seeing how his symptoms progressed, I think we could question if more could have been done earlier in the course of this patient’s disease - maybe when the initial abnormal, if not diagnostic, capillaroscopy results were available. Unfortunately, the patient not attending further clinic appointments reduced the opportunities to do this, but this also raised a good question about how we try to ensure patient engagement and deal with patients who don’t attend follow up.

**Key learning points/Conclusion:**

This was an excellent case for us to be able to learn about systemic sclerosis and the spectrum of disease it encompasses. It was interesting to learn about the different features and presentations of the disease, and how these different phenotypes correlate to certain antibodies classically being present, or as in this case, not present. It also enabled me to consider how we manage complex patients with multiple comorbidities and variable compliance. I feel that this conference would allow us to learn from other complex cases and how to best manage patients with similar issues, including systemic sclerosis, prominent pain issues, and gastrointestinal issues within rheumatology.

